# Transforming care for patients living with diabetes in rural Mexico: a qualitative study of patient and provider experiences and perceptions of shared medical appointments

**DOI:** 10.1080/16549716.2023.2215004

**Published:** 2023-05-31

**Authors:** Martha de Lourdes Arrieta-Canales, Joia Mukherjee, Hannah Gilbert, Hugo Flores, Melania Muñoz, Zeus Aranda, Samuel DiChiara, Carolina Noya

**Affiliations:** a Department of Global Health and Social Medicine, Harvard Medical School, Boston, MA, USA; b Department of Primary Care, Partners In Health Mexico/Compañeros En Salud, Ángel Albino Corzo, Mexico; c Division of Global Health Equity, Brigham and Woman’s Hospital, Boston, MA, USA; d Partners In Health, Boston, MA, USA; e El Colegio de la Frontera Sur, San Cristóbal de las Casas, Chiapas Mexico; f School of Public Health and Tropical Medicine, Tulane University, New Orleans, LA, USA; g Family Health Care Nursing, School of Nursing, University of California, San Francisco, CA, USA

**Keywords:** Chronic diseases, vulnerable populations, low- and middle-income countries, primary health care, community health, patient-centred care

## Abstract

**Background:**

Global prevalence of diabetes is increasing, causing widespread morbidity, mortality and increased healthcare costs. Providing quality care in a timely fashion to people with diabetes in low-resource settings can be challenging. In the underserved state of Chiapas, Mexico, which has some of the lowest diabetes detection and control rates in the country, there is a need to implement strategies that improve care for patients with diabetes. One such strategy is shared medical appointments (SMAs), a patient-centred approach that has proven effective in fostering patient engagement and comprehensive care delivery among underserved populations.

**Objective:**

This study aimed to understand the perceptions, experiences and insights of both patients living with diabetes and healthcare providers, who took part in a pilot SMA strategy implemented in five outpatient clinics in rural Chiapas.

**Methods:**

Following an exploratory qualitative approach, we conducted 50 in-depth interviews with patients and providers involved in diabetes SMAs and five focus group discussions with community health workers providing patient support and education.

**Results:**

The implementation of an SMA model changed how diabetes care is perceived, structured and delivered. Patients felt sheltered by group interactions based on trust, which allowed for the exchange of experiences, learning and increased engagement in treatment and lifestyle changes. Providers gained insights into their patients’ context and lived experiences, which resulted in improved rapport and quality of care. SMAs also restructured some operational aspects in the clinics and fostered the sharing of power and responsibilities amongst the staff.

**Conclusions:**

The SMAs model transformed care by providing a patient-centred, collaborative approach to diabetes care, education and support. Additionally, it reshaped the health-care team resulting in power-shifting and role-sharing among members of the interdisciplinary team. We therefore encourage decision-makers to expand the use of SMAs to improve care for patients with diabetes in low-resource settings.

## Background

The global prevalence of diabetes has increased steadily over the last four decades resulting in almost twice as many people living with the condition [1]. In 2019, estimated 463 million people were living with diabetes worldwide and 80% of them were in low- and middle-income countries (LMICs) [2]. In Mexico, 8.5 million people have the disease [3], which is the second leading cause of death in the country [4]. Contributing to the epidemic is a lack of knowledge about diabetes self-management [5]. The country declared diabetes a national health emergency in 2016 and has since sought to raise awareness of the disease, improve its prevention, detection and management, and to increase the quality of patient care [6].

Detection and control of diabetes are particularly challenging in rural areas of Mexico [7], where access to quality health services is limited. With most of the rural population working in the informal sector, citizens are often not covered by employment-based social security schemes [7], meaning that their only option for health care is the Ministry of Health facilities. Although Ministry of Health services are free of charge [8], there are several barriers that impede actual access to these facilities. For example, in the state of Chiapas, some rural facilities are not operational due to a lack of staff, medical equipment and supplies [9]. Even when facilities are operational, a large portion of the population has difficulty reaching them due to distance to health facilities, lack of public transportation and poor road conditions [10]. The majority (75.5%) of the region’s population lives in poverty [11]. The cost of transportation, in addition to the loss of earnings, makes it difficult for a large portion of the population to seek health services in a timely manner.

According to official statistics, the prevalence of diabetes in Chiapas (7.8%) is below the national average (10.3%) [12]. However, this is likely due to under-detection as the state has one of the lowest levels of screening in the country; in 2012, only 18% of the population was screened for diabetes in Chiapas compared to 26.2% nationally [13]. Although less stark, Chiapas also falls behind in terms of disease management, despite alarmingly low levels for indicators at the national level; 7.7% of people with diabetes (PWD) in the state had their levels of glycosylated haemoglobin (HbA1C) measured in the previous 12 months and 13.6% received a foot exam in the same time period, compared to 9.6% and 14.6% nationally [13,14]. Only 31% of PWD who are receiving treatment in Chiapas achieved optimal glycaemic control [15]. This level of clinical control falls far short of the Rule of Halves, according to which we would expect half of those receiving treatment to be under clinical control [16].

To address the diabetes epidemic in Mexico, innovative interventions are required to improve quality of life, achievement of clinical targets and improved quality of care for patients with diabetes. One promising approach is the Shared Medical Appointments (SMAs) care delivery model, which encourages patient education, active participation in care, lifestyle modifications and continuous support by prioritising interprofessional care in the context of peer support and organising long-term group-care visits with active discussions on relevant health-related topics [1,17,18]. SMAs have been shown to be a culturally sensitive approach to diabetes care, and to improve patient engagement and diabetes management [19,20]. This intervention has been shown to be effective in reducing key clinical indicators such as glycaemia, body-mass index and systolic blood pressure of PWD, and has been associated with improved perceptions and increased capacity for disease self-management amongst PWD [19,21–23].

Studies in the USA, some of which involved Latino patients, have demonstrated the efficacy of SMAs for the management of diabetes among low-income and underserved populations [24,25], providing evidence in support of expansion of this care delivery approach to low-income populations living in LMICs including Mexico. With this in mind, we decided to pilot the SMA intervention across five clinics in rural Chiapas, Mexico. This study aimed to understand the perceptions, experiences and insights of patients living with diabetes and healthcare providers, who took part in this pilot.

## Methods

### Study setting

Researchers from Harvard and the University of California-San Francisco partnered with Partners In Health Mexico/*Compañeros en Salud* (CES), a non-governmental organisation focused on improving healthcare delivery to underserved populations in the state of Chiapas. CES provides clinical and logistic support to 10 community-based rural clinics, providing health care to approximately 25,000 people [26]. The clinics are located in nine rural communities where agriculture (primarily coffee production) is the leading economic activity. The majority of the population are served by these clinics’ self-identify as non-indigenous (98%) and consider Spanish their first language. Educational attainment is low, with 35% of population completing secondary education, and 85% of the population live in poverty [27]. CES utilises a comprehensive approach to primary health care which includes training medical staff for non-communicable disease management, continuous delivery of medications, and coordinates a team of community health workers (CHWs). CHWs support patients with medication management and clinic visits as well as providing accompaniment and health education [28].

In 2017, SMAs were implemented across five CES-supported clinics. Staff organised sessions that lasted 2 h for groups of 8–12 patients with varied experiences of treatment and clinical management (Table 1). All patients had a preexisting diabetes diagnosis as defined by the Mexican Health Ministry Guidelines (two or more measurements of fasting blood glucose levels above 126 mg/dL). SMA groups met monthly for facilitated diabetes education and peer-support and medical evaluation. Nurses or CHWs led the sessions with support from a primary-care physician. Breakfast was served to the participants. This intervention was offered to patients, who chose enrolment, as an alternative to care-as-usual (monthly, 30 min one-on-one appointments with a physician).

**Table 1.** Comparison of the characteristics of the shared medical appointments for people living with diabetes versus care-as-usual in the compañeros En Salud-supported clinics.

Characteristic	SMAs	Care-as-usual
Providers	Physicians, nurses, CHWs	Physicians
Duration	2 hours: 30 minutes welcome and breakfast, 1.15 hours facilitated discussion and patient reviewing, simultaneous 1:1 medical check in and 15 minutes conclusion with simultaneous dispensing of medications.	15–30 minutes: discussion of symptoms and complaints, treatment adherence and medication adjustments.
Number of patients per session	8–12	1
Training required	Training as a physician, nurse or CHW. 2-hour session on SMA and group facilitation, 2-hour workshop on SMA, 3 SMA sessions under supervision of a trained clinical supervisor (nurse or physician).	Complete medical training. 3 months of supervised consultations to achieve desired proficiency in diagnosis and follow-up of patients living with diabetes.
Costs to be covered by the program	Medications Providers Breakfast	Medications Providers

CHWs: Community Health Workers; SMAs: Shared Medical Appointments.

### Sampling and data collection

For this qualitative exploratory study, we conducted in-depth interviews with patients (*n* = 38) and providers (seven physicians and five nurses), as well as five focus groups with CHWs (26 total participants). Inclusion criteria for patients included: 1) diabetes diagnosis; 2) older than 18; and 3) enrolled in SMAs for a minimum of 5 months. Exclusion criteria included: 1) inability to provide consent and 2) speech or hearing impediments. Inclusion criteria for providers (physicians, nurses and CHWs) included having experience providing services to patients with diabetes and participation in SMAs for a minimum of 5 months. All available providers were interviewed. Sampling utilised purposeful sampling techniques – a qualitative sampling strategy aimed at an in-depth understanding of a topic [29] – to secure an information-rich sample of patients with a wide range of contexts (i.e. treatment, clinical management and years since diagnosis). Providers were purposefully selected based on varying responsibilities and roles related to diabetes care (i.e. screening, diagnosis and follow-up, facilitation of SMA and conducting household visits). We provided participants with information about the study, including the principal investigator’s reasons for conducting the research, and offered them voluntary participation at monthly SMAs meetings. No providers or users declined to participate in the study or dropped-out. Informed verbal consent was obtained using procedures approved by Harvard’s Institutional Review Board and the Ethics Committee of the Chiapas Health Ministry.

Data were collected from August until December 2019 when saturation was reached. Interviews and focus groups were conducted onsite at participating clinics by the first author, a female general practitioner pursuing a master’s degree in global health delivery, with the help of a trained, local research assistant. The researchers conducting the interviews were part of the team working with CES and were therefore known to some of the participants, both in the interviews and in the focus groups. The interviews lasted approximately one hour in a private setting and were audio-recorded. In addition, the researchers took field notes. Interviews were conducted in Spanish and directly translated to English during transcription. The transcripts were not returned to the participants. Interviews followed semi-structured guides developed by the first author and a team of advisers with experience in service provision in rural areas, SMA and qualitative research, with variations based on type of participants. Patient interviews focused on the following topics: 1) participants’ experiences living with diabetes; 2) experiences with standard diabetes care in CES clinics; 3) experiences in SMAs; and 4) perception of the influence of SMA participation on treatment attitudes, self-efficacy, life-style modifications, and disease-related knowledge. Provider interviews focused on: 1) approach to diabetes care delivery; 2) SMA participation; 3) the process of SMA implementation; 4) influence of participation on care role and clinic operations; and 5) influence of patient participation on engagement and adherence to treatment. Interview guides can be found in supplementary file 1.

We conducted a focus group at each of the five participating clinics with CHWs to better understand their experiences related to the following topics: 1) diabetes service provision, 2) participation in SMAs, and 3) their perception on the influence of SMAs on patient engagement. Focus groups were conducted in Spanish, audio-recorded and transcriptions were translated to English. All available CHWs were invited to participate after one of the monthly SMA sessions. Focus group guides can be found in supplementary file 1.

Researchers analysed the qualitative datasets using a conventional content analysis approach [30]. Following a complete review of the dataset, a subset of interviews was open-coded independently by the first author and the local research assistant, which was subsequently edited for clarity and drafted by consensus between both researchers. The resultant codebook was applied to the entire dataset, which was coded by both researchers with support from Dedoose qualitative management software. Coded data were inductively examined to identify an initial set of key descriptive themes and subthemes which were then revised and refined through an iterative process to develop the final set of themes presented below. Participants did not provide feedback on the results. Authors adhered to the consolidated criteria for reporting qualitative studies (COREQ) 32-item checklist, which can be found in supplementary file 2.

## Results

Fifty interviews (38 patients and 12 providers) and 5 focus groups (26 total participants) were conducted. Patients attended between 5 and 24 SMA sessions, 68% were female and most (84.2%) were between 41 and 64 years of age (Table 2). Provider interviews included physicians (*n* = 7) and nurses (*n* = 5) with a mean of 5 years of experience caring for patients with diabetes; mean age for the providers was 28.8 years and 58.3% were female. The CHWs that participated in focus groups were mostly female (25 of 26 participants) with a mean age of 38.9 years.

**Table 2.** Sociodemographic characteristics of people with diabetes who participated in the study.

Characteristic		N (%)
Age in years	≤40	2 (5.3%)
	41–55	17 (44.7%)
	56–64	15 (39.5%)
	≥65	4 (10.5%)
Gender	Female	25 (68.4%)
	Male	13 (31.6%)
Treatment	Oral	29 (76.3%)
	Insulin	9 (23.7%)
Education (last completed level)	None	16 (42.1%)
	Elementary	17 (44.7%)
	Middle School	2 (5.3%)
	High School	1 (2.6%)
	Higher education	2 (5.3%)
Occupation	Farmer	9 (23.7%)
	Homemaker	25 (65.8%)
	Beekeeper	1(2.6%)
	Merchant	1 (2.6%)
	Teacher	1 (2.6%)
	Driver	1 (2.6%)
**Characteristic**	**Mean**	**Range**
Years since diagnosis	7.92	1–19
# of SMA attended	14.9	5–24
**Characteristic**	**Median**	**Range**
Yearly income (USD)	1,000	200–5,000

SMA: Shared Medical Appointment; USD: United States Dollar Multiple themes emerged from the data with an overarching theme of transformation. Themes are organised, below, according to transformations experienced by patients and by providers.

### Patient transformations

#### The SMAs allowed patients to feel more comfortable and build trust

Patients described the SMA environment as welcoming and friendly, which allowed them to socialise and talk about their personal experiences with other patients and the care team; these interactions made the appointments feel less formal. For example, one patient stated, ‘[It is] more entertaining … you get to greet them and chat while we are being examined and then we all can share our point of view’ (patient, female, 46 years old). Patients felt this helped them get to know other participants and the providers, which in turn also helped to build trust and cohesion. Patients explained that SMAs fostered a tightknit ‘group feeling’ that made diabetes care feel more like teamwork and less like an individual struggle. For example, a patient stated:

Here we give each other life, why? Because no one feels left out or discriminated against. No one tells you: “Hey you! Don’t do this!” No, here everything is up to you. It seems like a little, but it is really a lot that we can get here things that we are missing, like love and understanding. (Patient, male, 55 years)

#### SMAs led to improved support from peers and family

Patients described varying experiences of diabetes-related support, especially from family. There were often misunderstandings about their condition, as some described situations where family members actively discouraged patients from following dietary or treatment recommendations. Other participants described instances where symptoms were minimised, and some shared that they received no help or support from family in relation to medication adherence. For example, one patient stated, ‘We have no support, we can’t talk about these things [issues related to diabetes] at home. That is why we come to the clinic, so we can talk and receive counseling’ (patient, female, 51 years). In contrast, participants shared they felt supported when hearing about similar struggles other patients experienced, while also receiving advice and motivation.

Patients also described increases in support when they shared lessons learned from other patients with their families and invited relatives to their SMAs. One elderly patient with difficulty lowering his blood glucose detailed the experience of bringing his daughter-in-law to SMAs and how this led to support that improved adherence to treatment and diet. Another patient stated, ‘[SMAs help] a lot, because we can talk to our family about what we learned in the group and now we can do better what the doctor recommends’ (patient, female, 44 years).

#### Peers sharing insights and experiences encouraged lifestyle modifications and adherence to treatment

Patients reported sharing stories about complications such as hospitalisations or blindness. These stories served as cautionary tales for others in the group. Some patients mentioned that their peers’ real-life examples of success or failure provide anecdotal information they perceived as reliable. They cited success stories shared by patients around diet changes or improvement with new medications as motivation for change. For example, a patient stated ‘Now I can warn others, so it doesn’t happen to them [low blood sugar] … It can happen to anyone and maybe others are suffering from the same thing, but no one tells, and because we are not sharing, the others don’t know, and they suffer, and they can get worse’ (patient, male, 47 years).

Learning from experienced peers was described as especially helpful for newly diagnosed patients. These patients described receiving counselling and encouragement from patients who had more years living with the disease. They shared that being exposed to the experiences of others living with the disease helped in the process of disease acceptance and provided hope. One patient stated, ‘It is a very serious diagnosis [diabetes]; it can be seen as a death sentence. There is a lot of fear and denial. But at the SMAs … [we] can see other patients going through the same thing and can realize that there is treatment available and that they can keep their disease under control’ (patient, female, 25 years). The knowledge patients gained in SMAs resonated outside of the clinical space. Notably, patients explained that the lessons learned in SMAs translated into actions in their everyday lives. Patients noted several areas where they applied this knowledge, including making healthier dietary choices, committing to take short walks, trying new strategies for remembering medications, and accepting new forms of treatment such as insulin. For instance, one patient stated:

I started taking my medication and coming to the group, and with the things that I learned here, I started to have better control of my diet. I started eating fewer sugars; I was addicted to pastries, to cookies, to everything sweet, to candy … Coming to the SMAs has really helped me because now I don’t add sugar to my coffee, I eat very little pastries, and I try not to eat a lot of fatty foods. (Patient, female, 51 years)

### Provider transformations

#### SMAs encouraged trust-building between patients and the care team

Providers described how informal discussions with participants and the group environment helped foster a sense of trust between them and the patients. A key lesson was to remain judgement-free and respectful. Providers felt that SMAs fostered interactions where patients were more ‘authentic’ and were not as concerned with fulfiling providers’ expectations. Patients valued being encouraged to express their true beliefs around medications, such as insulin causing blindness. This allowed for active discussion as one physician noted:

[SMAs are] about sharing in a group, and that helps you feel less vulnerable when sharing doubts. Maybe you don’t feel comfortable expressing your doubts, but the person beside you is, and then you get this positive reinforcement that it is ok to have those doubts, that it is ok to ask. That helps diminish the gap that you see between patients and providers. (Physician, male, 28 years)

#### SMAs led to provider-insights and learning from patients’ lived experiences

When engaged in the SMAs, providers were exposed to patients’ experiences, and they described a shift towards viewing patients as leaders in their diabetes journey. Providers felt patients’ shared stories of treatment success provided credibility to their treatment recommendations. This, in turn, led to increased trust of clinical staff and uptake of treatment recommendations among patients. One physician explained:

Patients usually have concerns, especially around medications and especially around insulin or other treatments that are unfamiliar. But the fact that they can see someone from their own community … who is using a new treatment or doing something different or is taking other measures that they wouldn’t have considered before … It improves their confidence in the clinic and in the doctors … now someone they know is telling them the truth. So, little by little they get rid of their mistrust and fears. (Physician, female, 24 years)

Providers also felt they were part of the learning process, reporting valuable knowledge gained from listening to patients’ experiences. Providers explained that through SMAs they acquired a better understanding of the patients’ view of the disease, especially when they listened to how patients explained concepts to others. They learned keywords used by patients at sessions and incorporated these words into their own practice to better explain ideas and clarify concepts for others. For example, a physician stated:

Sometimes, you explain something to a patient, and they would explain to others in a different way. So, I took these ‘tools’ to better communicate with my patients. Sometimes the idioms they used were unfamiliar to me; I was not used to them, and understanding was hard. (Physician, male, 24 years)

Providers described newfound humility and compassion when approaching patients’ concerns, needs, and struggles. This led to tailoring treatment and lifestyle modifications to better fit patient context. A physician touches on this when stating, ‘Now, I know more about my patients … these things come up in the appointments, so then I can take that information and take all of it into account when prescribing treatment’ (physician, male, 24 years).

#### SMAs influenced changes in provider behavior

Providers often described using patient’s stories to better explain necessary treatment modifications or processes to patients that did not attend SMAs. For example, a CHW stated, “[SMAs are] very helpful because now when we do household visits, we have the patients’ lived experiences with us. We are not saying anymore: ‘This is what the doctor says…’ But instead: ‘Look, patients that have the same disease as you have shared this.’ That makes things easier” (CHW, female, 28 years).

#### Providers shifted responsibilities in SMAs to the role of facilitator

Providers explained that SMAs transformed their role from one of directing education to facilitating or guiding informational exchanges between patients. They explained that in SMAs, patients take the ‘lead,’ and physicians guide the educational messaging as needed. A physician stated:

You create a stronger bond with this kind of appointment [SMA] than with traditional appointments. You can break this barrier of being the doctor that sits on this side of the desk … and that the patient must follow. You can listen more to what patients have to say, and that is something that I keep using even outside SMAs. (Physician, male, 24 years)

Providers also mentioned patients gain confidence with their increased involvement, which led to patients setting an agenda aligned with their interests and concerns. Providers shared that after a couple of sessions, the topics and structure increasingly reflect the group’s curiosity. A CHW stated, ‘Before, we explained the subjects … but nowadays we just start the conversation, and they take the lead … They have learned to share with each other the things they are doing and what could be good for others’ (CHW, female, 29 years).

#### SMAs reduced time constraints observed in individual patient encounters

Providers explained that it was challenging for them to cover everything that is needed in traditional appointments which allot 20–30 min per patient. A physician stated, “You can’t really solve many things that are linked to the patients’ context … you can’t really know if the patients leave with a message they can really understand and … apply to daily situations” (physician, female, 24 years). In contrast, providers estimated that SMA sessions lasted 2 h and included 8–12 patients with about an hour dedicated to discussion and learning. Most providers felt care was more patient-centred in SMAs and as an additional benefit, it increased providers’ capacity to see other patients. A nurse mentioned, ‘It has become easier for us … SMA helps us to care effectively for our patients with diabetes without neglecting other kinds of patients’ (nurse, male, 49 years).

Even with efficiencies in care delivery, SMAs required time outside of sessions devoted to organisation and paperwork. Providers explained that to reduce patient load, SMAs would need to serve between 5 and 7 patients per session, which was a challenge during the harvest when many patients stay on the plantations that often prevents them from attending appointments at the clinic.

#### SMAs encouraged interprofessional care and ‘power-sharing’

Physicians shared their reflections on how SMAs promote a more prominent role for CHWs and nurses, where they engage and educate patients, lead sessions, and conduct portions of physical exams. Physicians also shared how delivering care as part of a team helps redistribute the burden of care by re-allocating responsibilities and fostering teamwork. One physician noted, ‘This changes the … dynamic and helps redistribute power … when we get nurses or CHWs to lead the sessions we are strengthening other parts of the team’ (physician, male, 28 years).

## Discussion

The implementation of SMAs in Chiapas restructured multiple aspects of care. Patients engaged with peers, learned from the group, improved their social support, and were able to make changes to their diabetes self-management techniques and care. Peer-to-peer learning, as documented previously [31,32], is fundamental to patient engagement in SMAs. Providers were able to gain valuable insights into the experiences of patients with diabetes and utilise this immersive experience to improve patient communication, engagement, and shared decision-making. In addition, SMAs helped with patient flow and patient load.

In line with previous studies [18,33], our analysis confirms that the SMA care delivery approach is unique in its ability to reshape the health provider-patient dynamic to enable truly patient-centred care. Learning from their peers helps patients feel more competent in managing their disease. Patient-centred interventions and patient empowerment are especially important when working with underserved populations, where traditional models of care tend to reproduce oppressive power dynamics leading to patient disengagement from care [34]. Allowing patients themselves to take the lead in the group sessions ensures that concerns affecting the patient population are addressed and that a safe environment is created for everyone to share their experiences and thoughts. As found previously [31,35], the SMAs space allows providers to learn about patients’ experiences and perceptions of their health status and to reflect on their own practice. Changes in power dynamics were also experienced between health professionals. SMAs were facilitated by CHWs and nurses, helping to foster interdisciplinary collaborative practice, as demonstrated in previous SMA interventions [36].

Although this study is unique in exploring the application of SMAs in LMICs, many of the findings are consistent with quantitative research conducted in underserved communities in the US. In particular, some of these studies reported decreased dissatisfaction with diabetes and diabetes distress, as well as improved diabetes-related quality of life, frequency of blood-sugar self-testing, dietary habits, and frequency of exercise among patients participating in SMAs versus those receiving individual care [25,37,38]. In other qualitative studies in the US, participants cited a sense of community, improved disease self-management, a sense of empowerment, motivation, increased trust in providers, and gaining knowledge from peers as positive aspects of this model of care delivery [18,39,40]. Our findings provide evidence that the SMA model can be effective in low-resource settings. Moreover, patients in our study did not report any of the negative aspects of SMAs found in studies conducted in the US, such as insufficient personal attention, logistical barriers, and loss of confidentiality suggesting that this model could be better suited to the cultural context of rural Chiapas [39].

Regarding healthcare providers, participants in our study highlighted similar benefits of SMAs to those reported in previous qualitative research, such as power-shifting, trust building, better information sharing, improved self-management, peer support, less repetition, and more efficiency than the 1:1 approach [18,40]. One concern raised by practitioners both in our study and in previous SMA interventions, was the administrative workload involved in arranging and facilitating the sessions [38,40]. However, all these comparisons must be considered with caution, as previous SMA interventions were implemented in urban settings in the US and were tailored to those circumstances. Cultural humility has been shown to be a key factor in the success of diabetes SMAs [20,24,41,42]. Thus, tailoring interventions to be culturally congruent is of upmost importance to support a successful implementation across different settings and sub-cultures. In our experience involving CHW from the local community was key in making this model successful in rural Chiapas.

This study is not without limitations. Since the interviewers were part of the team working with CES and the clinic staff, there is a possibility of social desirability bias. To reduce this bias, patients were assured of confidentiality and voluntary participation, while questions were designed to elicit sincere answers. Our study also offers some strengths, such as the participation of data collectors who were thoroughly trained in the use of qualitative interviewing, the adaptation of interviews to the local language, and the independent double coding of transcripts by two researchers. The research highlights the value of capturing and sharing patient and provider experiences of patient-centred strategies implemented in low-resource settings. This evidence can guide and inform providers in the implementation of patient-centred interventions, as well as serve as advocating for their increased implementation in LMICs. Further research is needed to assess clinical outcomes, quality of care, scale-up and feasibility of implementing SMAs for diabetes at a larger scale in Chiapas and other settings.

## Conclusions

This study shows how the SMA intervention may hold promise for low-resource settings, as it fosters patient-centred care and reconfigures patient-provider relationships, making it possible to counteract oppressive power dynamics associated with traditional models of care that particularly affect marginalised populations. SMAs foster peer-learning and peer-support, helping patients feel more comfortable and competent in managing their disease. This intervention meets the goals set forth in the new guidelines and trends for diabetes management, in which patient experience and self-reliance are the building blocks of quality health care. Implementing these models while working with underserved populations means bringing ‘state-of-the-art’ diabetes management to places that currently lack it.

## Paper context

Multiple studies have demonstrated the positive effects of shared medical appointments for vulnerable patients with chronic disease in the United States. However, implementation of this care delivery model in low- and middle-income countries has yet to be reported. Our study is the first to describe the experiences and perceptions of patients with diabetes and healthcare workers participating in shared medical appointments in low- and middle-income countries, specifically in rural Chiapas, Mexico, reporting many benefits.
